# Retraction: Dysferlin Interacts with Histone Deacetylase 6 and Increases alpha-Tubulin Acetylation

**DOI:** 10.1371/journal.pone.0192239

**Published:** 2018-01-29

**Authors:** The PLOS ONE Editors

The corresponding author, Michael Sinnreich, has requested retraction of this article because the data used in this study were found to have been manipulated, and the integrity of the study to be compromised.

The following issues were specified:

Images in Figures 1B and 3C had been duplicated;

Western blot images in Figure 2B (2nd row, NiNTA pulldown, IB: anti-FLAG) were found to be manipulated;

Western blot images in Figure 3D (1st row, IP: anti-alpha-tubulin, IB: anti-GFP) were found to be manipulated.

The University of Basel investigated this matter using its established procedures for matters of scientific integrity and found evidence of data manipulation.

In light of the concerns identified, the authors and the *PLOS ONE* Editors retract this article.

The journal editors have attempted to contact all co-authors on the manuscript by email. MS made the initial request for retraction. SDF, BAA and CT agreed with the retraction.
